# Home hospice for older people with advanced dementia: a pilot project

**DOI:** 10.1186/s13584-019-0304-x

**Published:** 2019-05-06

**Authors:** Shelley A. Sternberg, Ron Sabar, Glynis Katz, Ronit Segal, Liat Fux-Zach, Valerie Grupman, Gery Roth, Netta Cohen, Zorian Radomyslaski, Netta Bentur

**Affiliations:** 1 Israel Ministry of Health, Division of Geriatrics, 39 Yirmiyahu St, Jerusalem, Israel; 2 Sabar Health, Home Hospital and Hospice, Jerusalem, Israel; 3 Maccabi Healthcare Services, Tel Aviv, Israel; 4 EMDA – the Alzheimer’s Association of Israel, Jerusalem, Israel; 5 JDC-ESHEL – the Association for the Planning and Development of Services for the Aged, Jerusalem, Israel; 6 Tel Aviv University, Tel Aviv, Israel

**Keywords:** Advanced dementia, Palliative care, Hospice care, Homecare

## Abstract

**Background:**

Dementia is a terminal illness making the palliative and hospice approach to care appropriate for older people with advanced dementia.

**Objective:**

To examine clinical and health services outcomes of a quality improvement pilot project to provide home hospice care for older people with advanced dementia.

**Study design:**

Twenty older people with advanced dementia being treated in the Maccabi Healthcare Services homecare program, received home hospice care as an extension of their usual care for 6–7 months (or until they died) from a multidisciplinary team who were available 24/7. Family members were interviewed using validated questionnaires about symptom management, satisfaction with care, and caregiver burden. Hospitalizations prevented and medications discontinued, were determined by medical record review and team consensus.

**Findings:**

The findings are based on 112 months of care with an average of 5.6 (SD 1.6) months per participant. The participants were on average 83.5 (SD 8.6) years old, 70% women, in homecare for 2.8 (SD 2.0) years, had dementia for 5.6 (SD 3.6) years with multiple comorbidities, and had been hospitalized for an average of 14.0 (SD 18.1) days in the year prior to the project. Four patients were fed via artificial nutrition. During the pilot project, 4 patients died, 2 patients withdrew, 1 patient was transferred to a nursing home and 13 returned to their usual homecare program. The home hospice program lead to significant (*p* < 0.001)improvement in: symptom management (score of 33.8 on admission on the Volicer symptom management scale increased to 38.3 on discharge), in satisfaction with care (27.5 to 35.3,), and a significant decline in caregiver burden (12.1 to 1.4 on the Zarit Burden index). There were five hospitalizations, and 33 hospitalizations prevented, and an average of 2.1(SD 1.4) medications discontinued per participant. Family members reported that the professionalism and 24/7 availability of the staff provided the added value of the program.

**Conclusions:**

This pilot quality improvement project suggests that home hospice care for older people with advanced dementia can improve symptom management and caregiver satisfaction, while decreasing caregiver burden, preventing hospitalizations and discontinuing unnecessary medications. Identifying older people with advanced dementia with a 6 month prognosis remains a major challenge.

## Introduction

The last few decades have seen rapid growth in the population of older people living with dementia. Caring for older people with dementia places significant burdens on patients, families and society at large [1]. As the disease progresses, these demands increase significantly. Motor function, mobility, swallowing and communication are affected in addition to cognition in advanced disease. Older people with advanced dementia (OPAD) become totally dependent on others for their personal care and daily function [2]. Common complications of advanced dementia are eating and swallowing problems (86%), fever (53%), and pneumonia (41%) [2]. Six month mortality has been estimated at 25% and rises to 50% with an episode of pneumonia in the past [2]. The prognosis of advanced dementia is similar to widely metastatic breast cancer [3].

The palliative and hospice approach emphasizes comfort and care over invasive interventions. This approach while traditionally reserved for cancer patients has been advocated for advanced dementia. Research in the nursing home setting has shown improved quality of care and quality of life when a palliative approach is implemented for patients with advanced dementia and their families [[4–6].

Few studies have assessed home hospice for OPAD. In an Australian study of a dementia home hospice program, it was found that patients who had received usual care visited the emergency room 6.7 times more in the weeks before death than patients receiving palliative care [7]. A US study conducted in the community found that hospice care decreased the risk of hospitalization in the final month of life, improved pain control and shortness of breath, and increased caregiver satisfaction [8]. In Israel, 149,000 people over 65 were estimated to be suffering from dementia in 2010, with an expected increase to 210,000 by 2020 [9]. Most people with dementia in Israel live in the community (89%) and 31% of them are estimated to have advanced dementia [10].

The objectives of our quality improvement pilot project were to examine clinical and health services outcomes of a home hospice program for OPAD as an extension of usual homecare.

## Methods

### Design

A quality improvement pre-post project was implemented to expand existing Maccabi homecare services to include home hospice for OPAD.

### Setting

The multidisciplinary medical homecare program of one region of Maccabi Healthcare Services, the second largest HMO in Israel, with a regional population over 65 of 45,000 and 1200 medical homecare recipients. Hospice services are outsourced in this region and coordinated with the homecare program of Maccabi Healthcare Services.

### Target population

A convenience sample of 20 OPAD were identified as eligible for home hospice care by nurse case managers of the usual homecare program. This sample of 20 participants were eligible after reviewing 33 referrals. Thirteen subjects were deemed not to have advanced dementia. Criteria for eligibility were: 1) stage 7 or higher on the Global Deterioration Scale ie patients with profound cognitive deficits (inability to recognize family members), minimal verbal communication, total functional dependence, incontinence of urine and stool, and inability to ambulate independently [11]. 2) a family interested in the hospice approach to care and 3) the presence of a full time live in formal caregiver. Once identified, charts were reviewed by one of the authors (SS) for eligibility.

### The program elements

Two workshops were held for all staff to improve communication between teams, and to enhance knowledge about dementia and specific end of life dementia issues such as swallowing problems, hand feeding and managing behavioral problems. In addition, the essentials of palliative and hospice care were reviewed with an emphasis on defining goals of care, identifying suffering and pain, and legal frameworks.

After staff training, the home hospice program was implemented over the course of a year with a maximum of 6 months stay in the program per person. The 6 month maximum was defined by the limitations of the study budget and is a standard hospice stay as defined by US Medicare. The program included pre-planned visits by a physician at least once a month and by a nurse once a week. Both had specialist knowledge and skills in palliative care. Staff were available 24 h a day 7 days a week by telephone and made additional visits as needed. The team social worker visited on admission and then made contact by visiting or phone at least every 2 weeks. A spiritual care provider visited families deemed appropriate by the team. Every participant’s swallowing abilities were assessed by a speech and language pathologist when starting the program. These visits were more frequent than usual homecare where physician visits occurred every 3–6 months, nursing visits every 1–3 months, and social worker visits 1–2 times a year. Multidisciplinary team meetings were held every 2 weeks to review patient status and plans of care. Services such as occupational therapy, prescriptions or oxygen were provided as part of usual homecare.

During the first visit, the usual homecare nurse case manager and the head nurse of the home hospice program explained to the family the meaning of hospice care. They discussed families’ expectations and preferences, and those of the patient, and a care plan was created. During the planned visits, the hospice staff provided repeated instruction to families and formal caregivers about the objectives of hospice care, how to reach the team when needed and how to respond in difficult situations, e.g., breathlessness or extreme agitation. A kit was prepared for every home containing intravenous fluids and sets, medications, and oxygen, thereby making supplies readily available in the home. The social worker gave the family guidance on patients’ rights and social benefits, and provided ongoing caregiver support. The spiritual care provider met with family members and patients as deemed appropriate by the team.

### Outcome measures

Family members were interviewed at the beginning and end of the program. Tools developed and validated by Volicer et al. [12] for end of life care in advanced dementia were used to assess outcomes of the program. The tools included a symptom management measure (pain, breathlessness, depression, fear, anxiety, fragile skin, agitation, resistance and degree of calm) scoring range from 0 to 45 with higher scores reflecting better symptom management. The satisfaction with care tool was also used (0–33, higher scores reflecting greater satisfaction) [13]. The Zarit burden scale (0–88, higher scores reflecting greater burden) was used to assess caregiver burden [14].

To assess health services outcomes, we counted the number of medications that were deemed unnecessary by the medical team and then discontinued. One of us (SS) reviewed every chart on a regular basis. Every medical event such as a fever, infection, aspiration etc. was reviewed in detail. Every case where a potential hospitalization may have been prevented, was reviewed by SS, and the treating physician and nurse in order to reach consensus.

## Results

Of 33 patients thought to have advanced dementia by the homecare nurse case managers, 20 were found eligible for the home hospice program. Thirteen patients did not meet the study criteria for advanced dementia. The average age was 83.5 (SD 8.6) years and 70% were women. They suffered from multiple chronic illnesses: 65% had hypertension, 40% had ischemic heart disease, 30% were post-stroke, 30% had diabetes, 15% had COPD, and 15% had cancer. Four of the patients had a feeding tube – 2 with a nasogastric tube and 2 with a gastrostomy. On average, they had been in homecare for 2.8 (SD 2.0) years, 5.6 (SD 3.6) years with a diagnosis of dementia, and hospitalized for 14 days (SD 18.1) in the year preceding the project. None had advance directives, and 6 had a legal guardian (Table 1).

**Table 1:** Demographic characteristics of Home Hospice Program Participants (*N* = 20)

	*N*	% / Mean(SD)
Age (years)	20	83.5(8.6)
Female	14	70
Chronic Illness:		
Hypertension	13	65
Ischemic heart disease	8	40
Post-stroke	6	30
Diabetes	6	30
COPD	3	15
Cancer	3	15
Feeding tube	4	20
Years in homecare		2.8 (2.0)
Years with diagnosis of dementia		5.6 (3.6)

During 112 months of care with an average of 5.6 (SD 1.6) months per participant, 4 patients died – 3 at home and 1 in hospital. Two patients left the program because more aggressive treatment was requested by the family (1 subsequently died in hospital) and 1 patient was admitted to a nursing home. At the end of the 6 month pilot project, 13 out of the 20 patients returned to usual homecare.

A physician visited each patient on average 1.1 times a month and conducted an average of 0.6 telephone calls. Approximately 7% of the physician visits (9/121) and 25% of her phone calls (14/56) were outside of the regular working hours of the usual homecare program (8:00 a.m. to 4:00 p.m.). A nurse visited each of the patients on average 3.6 times per month and had an average of 0.8 phone calls. Fifteen percent of all her visits (63/413) and 31% of her phone calls (24/78) were outside of the regular working hours of the usual homecare. The social worker was involved in the care of 19 of the patients, conducted more than 50 visits, over 120 phone calls and participated in 15 family meetings. The spiritual care provider was involved in the care of 12 patients and their families and made 31 visits. She participated in 25 staff meetings and 15 family meetings.

Symptom management significantly improved from an average score of 33.8 at the start of the program to 38.3 at the end (*p* < 0.001). The main symptoms identified were pain, fragile skin, and agitation.

Family members’ satisfaction with care also significantly improved from an average score of 27.5 to 35.3 (*p* < 0.001). Caregiver burden significantly decreased from an average score of 12.1 to 1.4 (*p* < 0.001). (Table 2). Families described around the clock access to the team, and a high level of professionalism as being the most important added value of the program. In addition, all the families mentioned that the program gave them a sense of being supported and that they would recommend it to others. Most reported that they felt more comfortable caring for their loved one at home, that suffering had been decreased, and that they learned more about the trajectory of dementia. In addition, family members who met with the spiritual care provider, reported that the meetings contributed to their wellbeing and to that of the patient; meetings with the social worker contributed to reducing caregiver burden, to understanding the care plan, and to the process of returning to the usual homecare program.

**Table 2:** Outcomes of Home Hospice during a 6 month period of care

	Symptom management score (0-40)^*^	Family member satisfaction with care score (0-45)^*^	Family member burden of care score (0-88)^*^
T1	33.8	27.5	12.1
T2	38.3	35.3	1.4

^*^p < 0.001

On average 2.1(SD 1.4) medications were discontinued per person, 5 hospitalizations occurred, and 33 hospitalizations were prevented. The prevented hospitalizations included: 11 cases of aspiration pneumonia, 6 cases of urinary tract infection and 6 of cellulitis in 15 of the 20 OPAD.

## Discussion

This quality improvement pilot project of expanding usual homecare to include home hospice for OPAD improved symptom management and family satisfaction with care, reduced caregiver burden and unnecessary medication use, and prevented hospitalizations. Important elements of the program that may have contributed to the positive outcomes were: staff training and education, a dedicated multidisciplinary team specializing in palliative care, 24/7 staff availability, early and repeated discussions of care preferences, repeated instruction of families about what to expect and how to respond in different situations.

Implementing the pilot project also presented challenges. Firstly, thinking of dementia as a terminal illness appropriate for hospice care was a cultural shift for staff as well as for families. This required much education and reinforcement. Secondly, identifying OPAD with a 6 month prognosis was a significant challenge. At the end of the program, 13 patients were alive and returned to usual homecare. The mortality rate was 4/20 (20%) less than that predicted in the literature [2]. This prognostic uncertainty is born out in the literature. A report that examined seven studies of 6 month prognostication for OPAD found no consensus. The predominant criterion in all the studies was eating problems/malnutrition [15]. Most of the studies also included a decline in functional and cognitive status. Medicare uses the Functional Assessment Staging Tool (FAST), stage 7C whose criteria include: dependency in all ADL, incontinence, inability to say more than one word a day, lack of mobility, and at least one of the following in the previous year: aspiration pneumonia, pyelonephritis, septicemia, grade 3 or 4 bedsores, or eating problems causing the patient to eat or drink less than necessary to remain alive [16].

Another tool, the advanced dementia prognostic tool (ADEPT), which includes breathlessness, bedsores above grade 2, functional deterioration, bedbound, inadequate eating, fecal incontinence, poor BMI, weight loss and heart failure, was also found to have moderate predictive ability [17]. In this context, it is worth noting a tool developed by Aminoff in Israel, which includes 10 questions (agitation, shouting, pain, bedsores, malnutrition, eating problems, invasive action, unstable medical condition, suffering according to a professional, suffering according to a relative). A high score (7–9) predicts survival of about 1 month among OPAD in acute hospital wards. Prognosis in the home setting is not addressed by this tool [18].

Additional tools are also used to identify people in need of palliative care with mortality expected within a year. Two of these tools include prognostic criteria in dementia: the prognostic indicator guidance of the UK Gold Standards Framework and the NECPAL CCOMS-ICO, which was developed by the WHO Department of Health in collaboration with the Catalan Collaborating Centre for Palliative Care [19, 20].

Adapting these tools to enhance prognostication in advanced dementia will help kupot holim in identifying those OPAD with a 6 month prognosis, appropriate for hospice care.

This pilot project has significant limitations. Firstly, the project had a pre-post quality improvement design, small size and convenience sample. In addition, the study was done in only one homecare unit limiting its generalizability.

## Conclusions

Dementia is a terminal, incurable illness and therefore, palliative care is appropriate. The current quality improvement pilot project of adding hospice care to usual homecare showed improved symptom management and increased satisfaction of family caregivers with care, reduction in caregiver burden and in hospitalizations, and discontinuation of unnecessary medications. The challenges to implementing the program were the cultural shift involved in thinking of dementia as a terminal illness and identifying dementia patients with a 6 month prognosis appropriate for hospice care. A preliminary cost analysis done by Maccabi Healthcare Services found the program to be at least cost neutral, leading to a decision to include OPAD in Maccabi home hospice programs. The groundwork has been laid for other kupot to follow suit. In addition, the insights regarding the impact and challenges of implementing a home hospice dementia model will be valuable internationally. Although we were able to show decreased health services use and improved caregiver satisfaction, much research is still needed to define dementia patients with a poor prognosis.

## Abbreviations

BMI: Body Mass Index
COPD: Chronic Obstructive Pulmonary Disease
HMO: Health Maintenance Organization
NECPAL CCOMS-ICO: NECesidades PALiativas (Palliative Needs) World Health Organisation Collaborating Centre for Public Health Palliative Care Programmes of the Catalan Institute of Oncology
OPAD: Older People with Advanced Dementia
SD: Standard Deviation
UK: United Kingdom
US: United States
WHO: World Health Organisation
