# Correction to: home hospice for older people with advanced dementia: a pilot project

**DOI:** 10.1186/s13584-019-0329-1

**Published:** 2019-07-01

**Authors:** Shelley A. Sternberg, Ron Sabar, Glynis Katz, Ronit Segal, Liat Fux-Zach, Valeria Grofman, Gery Roth, Netta Cohen, Zorian Radomyslaski, Netta Bentur

**Affiliations:** 1 Israel Ministry of Health, Division of Geriatrics, 39 Yirmiyahu St, Jerusalem, Israel; 2 Sabar Health, Home Hospital and Hospice, Jerusalem, Israel; 3 Maccabi Healthcare Services, Tel Aviv, Israel; 4 EMDA – the Alzheimer’s Association of Israel, Jerusalem, Israel; 5 JDC-ESHEL – the Association for the Planning and Development of Services for the Aged, Jerusalem, Israel; 6 Tel Aviv University, Tel Aviv, Israel

**Correction to: Isr J Health Policy Res (2019) 8: 42**

**https://doi.org/10.1186/s13584-019-0304-x**

In the original version of this article [1], published on 6 May 2019, the name of author ‘Valeria Grofman’ was incorrect.

Originally the author name has been published as:

- Valerie Grupman

The correct author name is:

- Valeria Grofman

The original publication of this article has been corrected.
